# The role of sphingosine 1‐phosphate and its receptors in cardiovascular diseases

**DOI:** 10.1111/jcmm.15744

**Published:** 2020-08-17

**Authors:** Jie Ouyang, Zhihao Shu, Shuhua Chen, Hong Xiang, Hongwei Lu

**Affiliations:** ^1^ Center for Experimental Medical Research the Third Xiangya Hospital of Central South University Changsha China; ^2^ Department of Cardiology the Third Xiangya Hospital of Central South University Changsha China; ^3^ Department of Biochemistry School of Life Sciences of Central South University Changsha China

**Keywords:** cardiovascular disease, sphingosine 1‐phosphate, sphingosine 1‐phosphate receptor

## Abstract

There are many different types of cardiovascular diseases, which impose a huge economic burden due to their extremely high mortality rates, so it is necessary to explore the underlying mechanisms to achieve better supportive and curative care outcomes. Sphingosine 1‐phosphate (S1P) is a bioactive lipid mediator with paracrine and autocrine activities that acts through its cell surface S1P receptors (S1PRs) and intracellular signals. In the circulatory system, S1P is indispensable for both normal and disease conditions; however, there are very different views on its diverse roles, and its specific relevance to cardiovascular pathogenesis remains elusive. Here, we review the synthesis, release and functions of S1P, specifically detail the roles of S1P and S1PRs in some common cardiovascular diseases, and then address several controversial points, finally, we focus on the development of S1P‐based therapeutic approaches in cardiovascular diseases, such as the selective S1PR1 modulator amiselimod (MT‐1303) and the non‐selective S1PR1 and S1PR3 agonist fingolimod, which may provide valuable insights into potential therapeutic strategies for cardiovascular diseases.

## 1 INTRODUCTION

Sphingolipids are major components of cell membranes and play indispensible roles in the regulation of cellular functions such as cell growth, differentiation, ageing and death.^1^ Sphingosine 1‐phosphate (S1P) is a bioactive metabolite of sphingolipids. S1P and its receptors (S1PRs) are ubiquitously expressed.^2^

S1P has irreplaceable roles of the intracellular messenger and extracellular mediator in the body.^3^ In particular, SphK/S1P axis is involved in the development and functioning of the cardiovascular system. Most evidence supported a cardioprotective role for S1P, such as fine‐tuning cardiac contractility and heart rate through the S1PRs signalling pathway.^4^

Given the importance of S1P for human health, many S1P mimics have been synthesized, notably fingolimod, which have been under clinical investigation for treating neurodegenerative diseases such as multiple sclerosis.^2^ Though fingolimod efficacy seems promising so that fuels further evaluation, its benefits should be weighed against off‐target toxicity that mainly occurs in the cardiovascular system. Owing to the significance of the S1P‐S1PRs axis in cardiovascular physiology and pathophysiology, it has attracted increasing interest inside cardiovascular research.

## 2 S1P GENERATION AND FUNCTION

### 2.1 S1P Biosynthesis

Sphingosine 1‐phosphate is a biological active mediator derived from sphingosine, the backbone of most natural sphingolipids.^5^ So far, many different kinds of cells have been found to be capable of synthesizing and releasing S1P into the circulation. It is generally believed that the major source of plasma S1P is platelets, because this cell type relatively lacks S1P‐degradating enzymes which are required for maintaining intracellular levels of S1P.^6^ However, recent evidence suggested that the contribution of platelets to plasma S1P is not significant or might be limited because platelet depletion, by infusion of an anti‐GPIb antibody, did not profoundly reduce the circulating levels of S1P.^7^ In other words, platelets are unlikely to be the primary source of plasma S1P under physiological conditions. Stronger evidence has shown that endothelial cells (ECs) contribute significantly to plasma S1P. Venkataraman and colleagues observed that in vitro, laminar shear forces can reduce the expression of S1P‐degrading enzymes S1PL and S1P phosphatase‐1 (Sgpp1) and more importantly, simultaneously stimulate ECs to secrete S1P,^8^ leading to constitutive release of S1P from ECs with the help of the spinster‐2 transporter (Spns2).^9^ In addition, red blood cells and neutrophils are also plasma S1P providers.^3^, ^10^, ^11^

**FIGURE 1:**
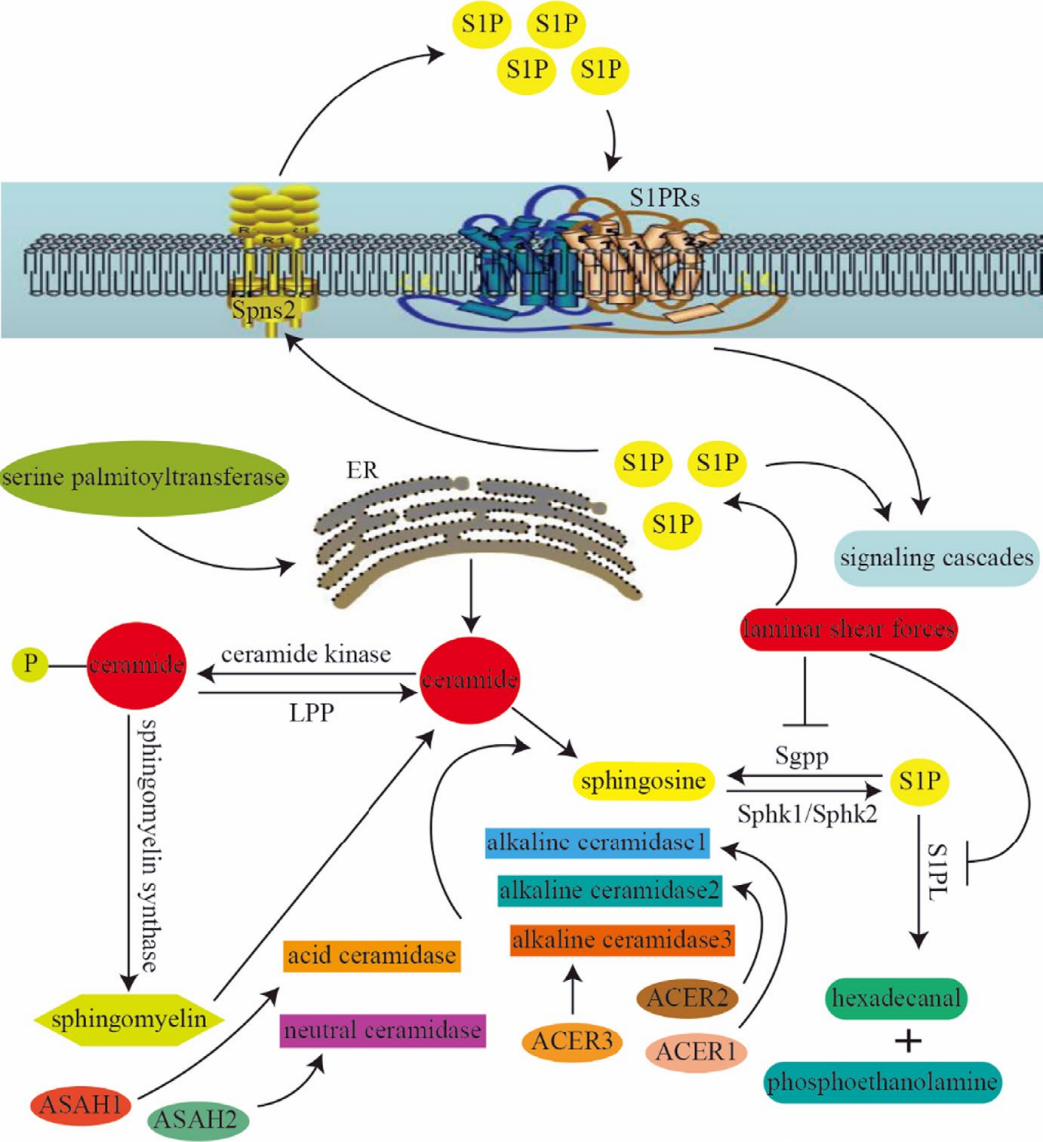
The biosynthesis and secretion of S1P. ECs, endothelial cells; ER, endoplasmic reticulum; LPP, lipid phosphatase; RBCs, red blood cells; S1P, sphingosine 1‐phosphate; S1PL, S1P lyase; S1PRs, sphingosine 1‐phosphate receptors; Sgpp, S1P phosphatase; Sphk, sphingosine kinase; Spns2, spinster‐2 transporter

Like other sphingolipids, S1P is produced by a sequence of enzyme‐mediated reactions. The de novo synthesis of S1P is initiated in the endoplasmic reticulum by serine palmitoyltransferase to produce ceramide, which can also be formed during the degradation of complex sphingolipids.^12^ Ceramide is deacylated by ceramidase into sphingosine,^13^ which is, in turn, phosphorylated by sphingosine kinase (SphK) isoenzymes, including SphK1 and SphK2, to generate the S1P.^5^ In this catabolic process, ceramidases are key enzymes, including acid ceramidase, neutral ceramidase, and alkaline ceramidase 1, 2 and 3, which are encoded by five different genes, ASAH1, ASAH2, ACER1, ACER2 and ACER3, respectively.^14^ Ceramides can be phosphorylated by ceramide kinase to become ceramide‐phosphate, which may be recycled back to ceramide by lipid phosphatase or used by sphingomyelin synthase to synthesize sphingomyelin. Additionally, S1P degradation is also important for maintaining its circulating levels.^15^ S1P can be dephosphorylated and converted to sphingosine by Sgpp or irreversibly degraded to hexadecanal and phosphoethanolamine by S1PL (Figure 1).^16^

### 2.2 S1P Functions in heart and blood vessels

Human S1P plasma levels range from 0.1 to 1.2 μmol/L with a half‐life of approximately 15 minutes,^8^ indicating active biosynthesis and turnover for S1P. Under normal and pathological conditions, S1P exerts its bioactive functions by interacting with cell membrane targets such as G‐protein‐coupled S1P receptors (S1PR1‐5) and intracellular signals, which regulate downstream effectors and a large variety of cellular functions (Figure 1).^17^ Varying S1P plasma concentrations are closely related to multiple cardiovascular diseases, including heart rate change, coronary heart disease (CAD), atherosclerosis, heart failure and myocardial infarction (MI).^18^, ^19^, ^20^, ^21^ Therefore, the S1PL inhibitor, 6‐[(2R)‐4‐(4‐benzyl‐7‐chlorophthalazin‐1‐yl)‐2‐methylpiperazin‐1‐yl] pyridine‐3‐carbonitrile, has been applied to increase S1P plasma concentrations and slow down heart rate and consequently improve heart function.^18^ In patients with CAD, the severity of stenosis is positively associated with the increased serum S1P concentrations. Multivariate logistic regression analysis has shown that serum S1P was more predictive of obstructive CAD than those conventional risk factors such as age, sex, family history of CAD and hypertension, with an odds ratio of 7.61.^20^ In low‐density lipoprotein receptor‐deficient (LDL−/−) mice, elevating endogenous S1P levels could significantly inhibit the development of atherosclerosis by reducing plaque formation, macrophages content and lipopolysaccharide‐induced recruitment of monocytes into the peritoneal cavity, leucocyte adhesion to capillary walls and endothelial permeability.^22^ Moreover, serum S1P was found to be inversely associated with peripheral arterial disease and carotid stenosis in humans, and this may be more accurate for predicting these diseases than HDL.^19^ Also, in patients with heart failure, plasma S1P levels have a negative correlation with the severity of heart failure. Patients with severely reduced left ventricular ejection fraction (LVEF < 40%) have lower plasma S1P levels than those with mildly reduced LVEF (LVEF > 40%), and the plasma S1P of NYHA class III and class IV patients is obviously lower than that of NYHA class I and class II patients.^23^ Besides, in patients with acute MI, plasma S1P showed a transient increase and subsequently gradual decrease within 48 h.^24^ S1P can be released at sites of tissue injury in the heart and thus protects against myocardial ischaemia‐reperfusion injury.^25^ Even S1P can be increased in a transient myocardial ischaemia caused by percutaneous coronary intervention, which gradually returns to normal 12 h later. This rapid and transient increase of S1P provides cardioprotection against ischaemic cell injury through the activation of the Pak1/Akt/eNOS signalling pathway.^26^

## 3 S1P‐S1PRs SIGNALLING IN CARDIOVASCULAR DISEASES

### 3.1 The dual role of S1P and S1PRs in Myocardial Infarction

Myocardial infarction (MI) is the death of myocardial cells due to ischaemia, which results from the imbalance between blood oxygen demand and supply. It can present as common discomforts in the chest, upper extremity, lower jaw and upper abdomen during exertion or at rest, accompanied by dyspnoea, sweating, nausea and syncope.^27^ Although the current understanding of MI is relatively comprehensive, it is still of significance for exploring the underlying mechanisms for better clinical management.

Myocardial infarction involves inflammatory response, and S1P is a critical regulator of immune inflammatory response, so the role of S1P in MI has been extensively determined. S1PR1‐3 is the main isoforms of S1PRs expressed in the heart,^28^, ^29^ where they regulate cardiac contractility and calcium handling, among other regulatory activities.^29^ Different S1PRs have distinct regulatory effects, and the distribution and composition of these receptor subtypes in the heart directly reflect differential regulatory effects and cardiac performance. As Landeen et al described, S1PR1 is predominantly expressed in cardiomyocytes, while S1PR3 is the most common subtype in fibroblasts.^30^

**FIGURE 2:**
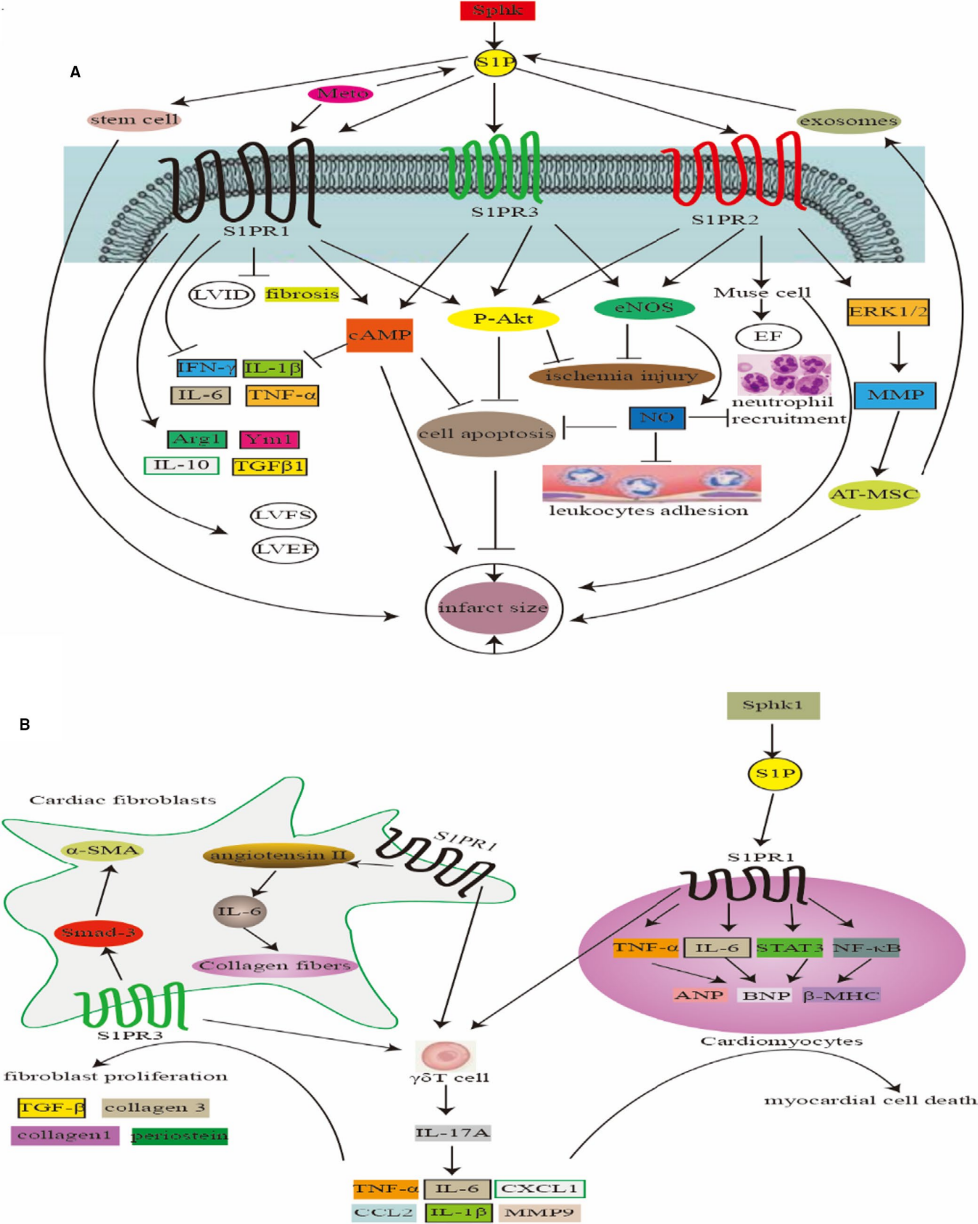
Differential effects of S1PRs at different stages after myocardial infarction. A, A protective effect of S1P in myocardial infarction in the early stage. B, A damaging effect of S1P in myocardial infarction in the late stage. EF, ejection fraction; LVEF, left ventricular ejection fraction; LVFS, left ventricular fraction shortening; LVID, left ventricular internal dimension; Meto, metoprolol; S1PR, sphingosine 1‐phosphate receptor

Both S1PR2 and S1PR3 can protect the myocardium from ischaemia‐reperfusion injury, which is attributed to Akt phosphorylation, eNOS activation and NO release.^29^ Compared with healthy individual, MI patients have lower levels of plasma HDL‐bound S1P,^31^ which may affect S1PR2‐3 activation and cause cardiac dysfunction. In animal model, SphK activity was significantly reduced in uninfarcted myocardium (RM) in the first 2 weeks after MI, but oral SEW2871, a selective agonist of the S1PR1, effectively reduced RM‐induced apoptosis by restoring Akt phosphorylation levels.^32^ Moreover, S1P shows robust inhibition of inflammatory neutrophil recruitment and cardiomyocyte apoptosis through S1PR3‐mediated NO pathway, which can reduce the infarct size by 40% and effectively inhibit the adhesion of leucocytes to activated endothelium.^33^ The role of C1q/TNF‐related protein‐1 in reducing the MI area, myocardial cell apoptosis and pro‐inflammatory gene (eg TNF‐α, IL‐6 and IL‐1β) expression is also tightly related to S1P signalling, mainly S1PR1‐ and S1PR3‐mediated pathways, which activates cAMP signalling, thereby preventing myocardial ischaemia.^34^ Also, S1P may stimulate tissue regeneration by attracting hematopoietic stem cells to the infarct site, improving heart function.^35^ After acute MI, the S1P‐S1PR2 pathway mediates the homing of bone marrow‐derived Muse cells to the damaged heart to reduce the infarct size and increase ejection fraction (EF), consequently help restore cardiac function.^36^ Similarly, in the C57BL/6J mouse MI model, S1P pre‐treatment significantly enhanced the migration and anti‐apoptotic effects of adipose tissue‐derived mesenchymal stem cells (AT‐MSC). S1P promotes AT‐MSC migration through activating the ERK1/2‐MMP‐9 pathway and protects AT‐MSC from apoptosis through Akt activation, both of which are mediated by S1PR2.^37^ Interestingly, the exosomes secreted by AT‐MSC are also involved in myocardial repair through the S1P/SphK1/S1PR1 signalling pathway. AT‐MSC‐secreted exosomes could improve left ventricular ejection fraction (LVEF) and left ventricular fraction shortening (LVFS), reduce left ventricular internal dimension (LVID), cardiac fibrosis and apoptosis after MI, and reverse MI‐induced M1 polarization of macrophages and promote the M2 polarization of macrophages. M1 macrophage polarization is manifested by the increased production of IL‐6, IL‐1β, IFN‐γ and TNF‐α while M2 macrophage polarization is represented by the increases expression of Arg1, Ym1, TGFβ1 and IL‐10.^38^ Additionally, the beneficial effect of metoprolol (Meto) on delaying the progression of heart failure after MI should be partially ascribed to impact S1P signalling. Meto was shown to prevent catecholamine‐induced S1PR1 down‐regulation and promote β3AR‐dependent S1P secretion/signalling (Figure 2A).^39^

Cardiac fibroblasts are important in maintaining normal cardiac function and are also known to participate in cardiac repair and dysfunction after MI. Excessive cardiac fibroblasts is a major issue in heart failure, which reduces the heart contraction strength and impairs its diastolic capacity. The S1PR3, highly expressed in fibroblasts, has an enhancing role in inducing cardiac fibrosis, mainly promoting the expression of α‐smooth muscle actin (α‐SMA) through Smad‐3.^40^ Besides, overexpression of S1PR1 has a promoting effect on myocardial fibroblasts for remodelling, mainly through the production of angiotensin II and IL‐6, thereby increasing the accumulation of collagen fibres.^41^ This seems inconsistent with the notion that S1P benefits myocardial function in MI. Nevertheless, the accumulated data have shown that cardiac S1P promotes cardiac remodelling during chronic cardiac inflammation. Zhang et al found that after MI, cardiac S1P was increased, and the SphK1/S1P/S1PR1 signal in myocardium was amplified, accompanied by the presence of pro‐inflammatory transcription factors NF‐κB and STAT3 and pro‐inflammatory cytokines TNF‐α and IL‐6 as well as then the up‐regulation of remodelling genes ANP, BNP and β‐MHC, indicating that the SphK1/S1P/S1PR1 signalling activates myocardial inflammation and exacerbates cardiac remodelling and dysfunction post‐MI.^42^ Furthermore, during left ventricular remodelling after MI, S1PR1 and S1PR3 promote the recruitment of γδT cells to the infarcted heart to produce IL‐17A and then stimulate macrophages to produce pro‐inflammatory cytokines such as TNF‐α, IL‐6, IL‐1β, CCL2, MMP9 and CXCL1, thereby aggravating myocardial cell death and fibroblast proliferation, indicated by the increased expression of fibrosis genes such as TGF‐β, collagen 1, collagen 3 and periostein (Figure 2B).^43^

Taken together, the molecular regulatory mechanism of S1P in MI is not completely clear. S1P may have a dual role depending on the phases of MI and cardiac cell types (myocardial cells and fibroblasts) involved. In the early stage, S1P plays a protective role while during the later stage, it promotes myocardial remodelling. The mechanism and treatment of MI warrants further investigation.

### 3.2 The protective effect of S1P and S1PRs against myocarditis

Myocarditis is an inflammatory disease of myocardial cells. It can be identified by conventional histology and immunohistochemical techniques as monocyte infiltration into the myocardium. Myocarditis may be local or diffuse and can be divided into acute, subacute and chronic phase.^44^ Myocarditis is primarily caused by virus infection, particularly the Coxsackie virus, including group B‐2‐5 and group A‐9.

Although the link between S1P and myocarditis is not well defined, it has been reported the involvement of S1P signalling in myocarditis. As a blood‐borne inflammatory mediator, S1P showed an inhibitory effect on myocarditis caused by Coxsackievirus B3. S1P can enhance anti‐inflammatory and anti‐myocarditis effects by up‐regulating invariant natural killer T cells in the spleen and left ventricle blood and concomitantly reducing virus capsid protein expression and apoptosis in the myocardium.^45^ This anti‐myocarditis activity of S1P was further confirmed by Kitabayashi et al who observed that the activation of S1PR1 and S1PR3 by the non‐selective agonist fingolimod prevented the development of experimental autoimmune myocarditis in rats, and fingolimod was more effective in this aspect than the immunosuppressive drug tacrolimus,^46^ proving the therapeutic potential of S1P in human myocarditis.

### 3.3 Differential effects of S1P and S1PRs on different types of cells in Vascular System

Regarding the role of S1P‐S1PRs in blood vessels, the general consensus is that S1P performs different pathophysiological functions depending on the types and expression levels of different receptors in different cells. Under physiological conditions, all S1PR1‐3 subtypes are expressed in ECs, but S1PR1 is the predominant one regulating ECs functions, including proliferation and migration. In ECs, the vasorelaxing effect of S1P is mediated by S1PR1, which stimulates the PI3K/Akt/eNOS pathway and results in subsequent NO production,^47^ by the same mechanism, S1PR1 also protects ECs from H2O2‐induced apoptosis and caspase‐3 activation.^48^, ^49^ As known, oestrogen is a beneficial factor for the cardiovascular system because it can induce vasodilation through eNOS activation and NO release, which is consistent with the vasodilator effect of S1P. In fact, S1PRs are involved in oestrogen‐induced vascular effects. The reduction in S1P‐mediated eNOS activation during aging is one of the mechanisms for endothelial dysfunction, but oestrogen can restore the expression of S1PR1 in the body.^50^ In addition, Sukocheva et al reported that oestrogen may be a regulatory factor upstream of S1P. They observed that 17β‐estradiol (E2) treatment of ECs results in rapid, transient and dose‐dependent increase in SphK activity and S1P production, accompanied by increased levels of cytosolic S1PR1, which induces Akt/eNOS activation.^51^ It seems to suggest that the protective effect of oestrogen may partly attribute to the S1P. Generally by coupling to different intracellular pathways, S1P/S1PR1 exerts a multitude of cellular functions. Studies have shown that S1P‐mediated endothelial integrity is mediated by the S1PR1‐Gαi‐Cdc42 pathway. S1P promotes cell proliferation and endothelial barrier function through the S1PR1‐Gαi‐Rac1 and S1PR1‐Gαi‐Cdc42 pathways. Upon inhibition of Gαi‐mediated signalling, S1PR2‐Gα12/13‐RhoA pathway can be harnessed to induce cell contraction and barrier function loss.^52^

**FIGURE 3:**
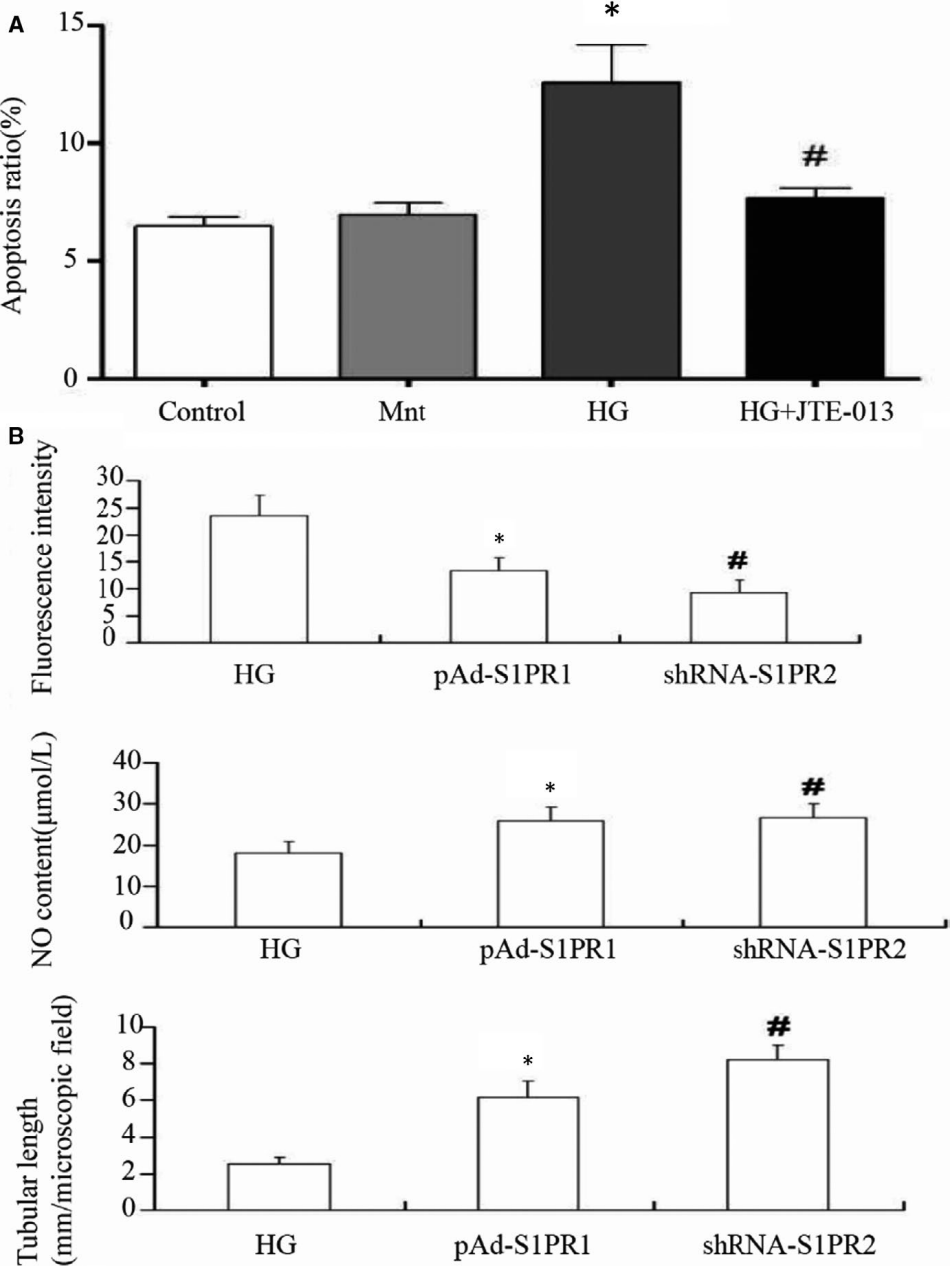
S1PR2 participates in endothelial mitochondrial apoptosis and dysfunction. A, S1PR2 antagonist inhibits HG‐induced cell apoptosis. After 1 h of exposure to 1 mmol/L JTE‐013, HUVECs were treated with normal glucose, high mannitol and high glucose for 72 h. *P < 0.05 vs the control group. ^#^ *P* < 0.05 vs the HG group. B, S1PR1 reduces, but S1PR2 enhances hyperglycaemia‐induced oxidative stress and morphogenetic response in HUVECs. The cells were transfected with either pAd‐S1PR1 or shRNA‐S1PR2 for overexpressing S1PR1 and silencing S1PR2, respectively. *, ^#^ *P* < 0.05 vs the HG group. Adopted with permission from Ref.^54^, ^55^ HG, high glucose; Mnt, mannitol

However, different S1PR subtypes may exhibit differential and even opposite biological effects. We reported that in human umbilical vein endothelial cells (HUVEC), high glucose causes mitochondrial apoptosis (Figure 3A), which is an early manifestation and important factor of vascular complications,^53^ and can be partially reversed by the S1PR2 receptor antagonist JTE013. In ECs, high glucose condition resulted in increased expression of S1PR2, but not S1PR1 and S1PR3, leading to mitochondrial apoptosis through the Akt/GSK‐3β signalling pathway.^54^ Overexpressing S1PR1 and silencing S1PR2 have a similar effect on reversing high glucose‐induced EC damage, indicated by reduced reactive oxygen species, increased NO and morphogenetic reversal,^55^ suggesting that S1PR1 and S1PR2 have opposite effects on ECs (Figure 3B). In agreement with these findings, other studies observed that pharmacological blockade of S1PR2 using JTE013 had a protective effect against oxidative stress‐induced brain ECs permeability by inhibiting p38 and ERK1/2‐dependent cPLA2 phosphorylation and activation.^56^

Unlike ECs with S1PR1 as a key regulator, most of smooth muscle cells express S1PR2 and S1PR3 and make good use of S1PR2 for protective effect. In smooth muscle cells, S1PR2 may act antagonistically to the promoting role of S1P in vascular proliferation, survival and differentiation. Activation of S1PR2 leads to decreased proliferation and migration of smooth muscle cells,^48^ which is considered to be involved in accelerating the pathological processes in the development of cardiovascular diseases, such as atherosclerosis. In line with this, Li et al have demonstrated that S1P can induce epicardial progenitor cells (EpiCs) differentiation into coronary vascular smooth muscle cells, chiefly through its receptors S1PR3 and S1PR2, but not S1PR1,^57^ indicating the importance of S1P signalling in vascular differentiation and maturation. This finding provided insights into the pathogenesis of hereditary cardiovascular dysplasia and gives a clue to explain why smooth muscle cells retain differentiated under normal circumstances. By contrast, S1PR1 can cause damage to smooth muscle cells. For instance, Ang II‐mediated APE/Ref‐1 translocation stimulates S1PR1 expression in VSMC, resulting in vascular dysfunction.^58^

As shown above, S1P shows numerous, and often opposite, regulatory functions in two distinct cell subtypes even in the same tissue such as blood vessels, which is primarily due to S1PR multiplicity, differential expression and cell specificity. This reminds us to take into considerations the pleiotropic effects of SIPRs when developing targeted drugs.

### 3.4 The opposite effect of S1P and S1PRs in hypertension

Hypertension is one of the leading risk factors for cardiovascular complications such as coronary heart disease, stroke and cognitive dysfunction.^59^ Hypertension is closely connected with altered microvascular function and structure and activated immune system.^60^ At present, hypertension is thought to be caused by the complex interactions of genetic and environmental factors and is related to a higher RAAS system activation. Of course, other multiple (nervous, endocrine and circulatory) systems are also involved in the pathology of hypertension.

NO is the main endothelium‐derived vasoactive factor that regulates blood pressure (BP), and S1P is a potent activator of eNOS through high‐affinity G‐protein‐coupled receptors. S1P can effectively reduce BP by activating S1PR1‐mediated release of the vasodilatory factor NO. This is evidenced that in angiotensin II mouse model, functional S1PR1 antagonism using fingolimod‐reduced S1PR1 expression in mouse endothelium and thus exacerbated hypertension, highlighting the potential harmful effects of fingolimod on vascular function.^61^ In addition, Swendeman et al suggest that ApoM‐Fc, a soluble carrier for S1P, activates S1PRs in a steady manner without altering the number of circulating lymphocytes. ApoM‐Fc‐bound S1P shows more potent reduction of BP in hypertensive mice and myocardial damage after ischaemia/reperfusion injury.^62^ In pulmonary arterial hypertension, S1P‐treated mesenchymal stem cells (MSCs) obviously reduced the right ventricular systolic BP and also cause a significant reduction in the right ventricular weight ratio and pulmonary vascular wall thickness.^63^ Furthermore, part of the increase in blood pressure caused by oestrogen deficiency is due to S1P. In ovariectomized female adult SD rats, it was found that SphK1/2 activity, S1P level and S1PR1 expression were all significantly reduced, but S1PR2 expression as well as systolic, diastolic and pulse pressures were obviously increased.^64^ All these collected data support the mighty role of S1P in lowering BP and mainly related to S1PR1.

However, some studies have reached the opposite conclusion. They found that S1P may promote the development of hypertension instead. Meissner et al have found that the involvement of the S1P signalling axis in AngII‐induced BP increase is related to increased circulating T cell counts via the regulatory mechanism involving hematopoietic SphK2, but not Sphk1.^60^ Other studies have added evidence shown that S1P chemotaxis and T cell mobilization are key factors for the onset of experimental hypertension.^65^ S1PR activation by fingolimod produced moderate hypertension (2‐3 mm Hg) in clinical setting and caused dose‐dependent hypertension by activating S1PR3 in rats.^66^ In addition, in AngII‐induced hypertension, inhibition of SphK1 attenuated the second stage of transmembrane Ca2+ influx. Similarly, external application of S1P could trigger store‐operated calcium channels (SOC)‐dependent Ca2+ influx, and SphK1 gene deletion significantly inhibited the acute hypertensive response of anaesthetized mice to AngII and the sustained hypertensive response of conscious animals to continuous infusion of AngII.^67^

Collectively, the differential regulatory effects of S1P on hypertension may be determined by multiple variables, such as the presence of different receptor subtypes, S1P isoforms and SphK isoenzymes. Typically, S1PR1 serves a role in controlling BP by activating NO release, while the activation of S1PR3 will increase BP. S1P‐binding ApoM‐Fc reduces hypertension while increased hematopoietic SphKs activity promotes hypertension.

### 3.5 The dual role of S1P and S1PRS in atherosclerosis

Atherosclerosis is a chronic arterial occlusive disease in which both innate and adaptive immune mechanisms are involved. Inflammation is a key culprit in all stages of atherosclerosis,^68^ and specially, inflammation and EC activation drive the initiation of atherosclerosis. Activated ECs and up‐regulated endothelial cell adhesion molecules (ECAM) mediate the focal recruitment and attachment of monocytes and lymphocytes to vessel walls, where they penetrate the intima and trigger inflammation.^69^ What's more, vascular smooth muscle cells (VSMCs) are also involved in the initiation and progression of atherosclerosis once activated by growth factors and inflammatory cytokines. Activated VSMCs rapidly proliferate and then migrate to the intima to produce various growth factors including the vascular endothelial growth factor and the platelet‐derived growth factor (PDGF) and most importantly, contribute to different plaque cell phenotypes and generation of a large quantity of extracellular matrix (ECM), eventually resulting in vessel wall thickening and luminal narrowing.^70^

**FIGURE 4:**
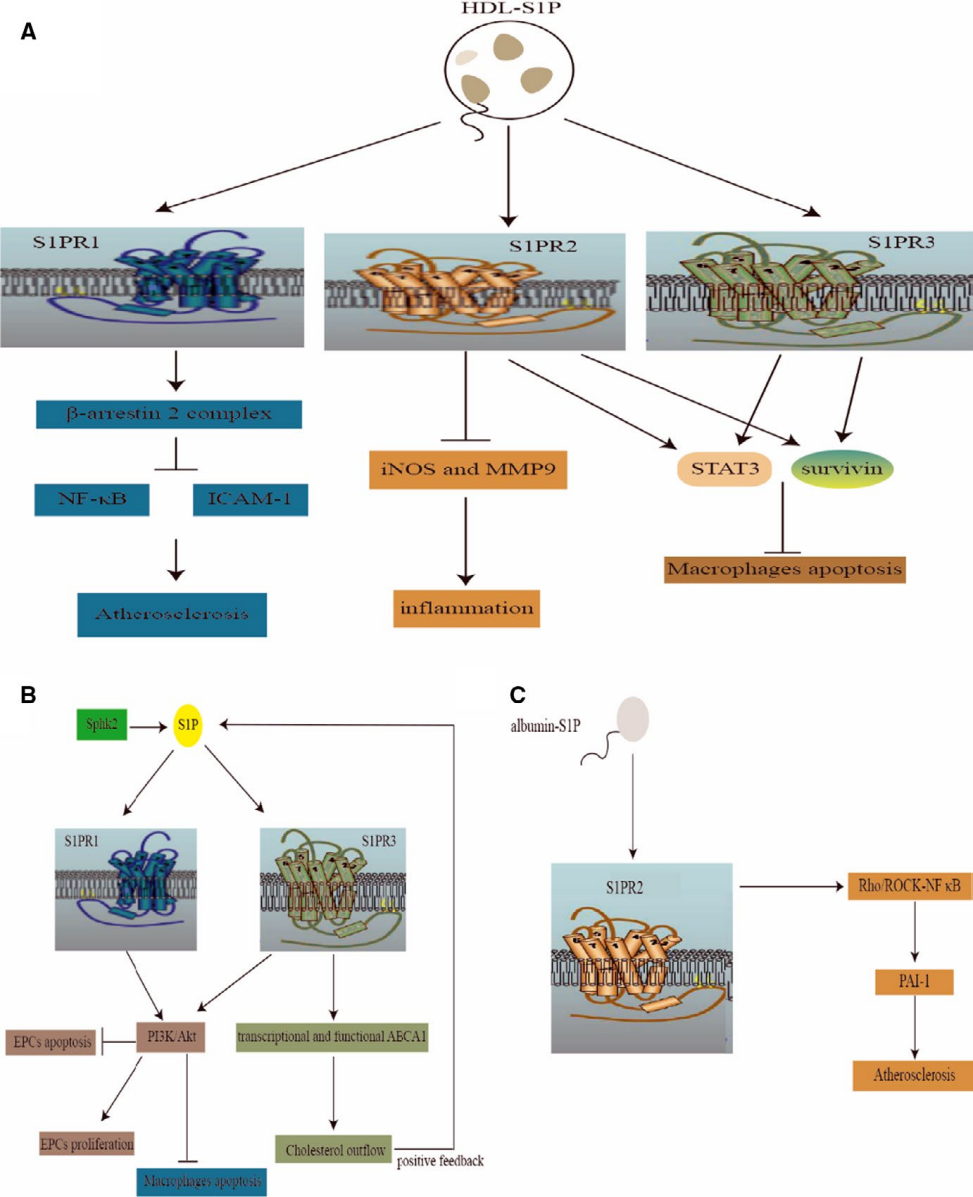
The multifaceted role of S1P in atherosclerosis. A, HDL‐S1P has anti‐atherosclerotic and anti‐inflammatory effects and inhibits macrophage apoptosis. B, S1P promotes endothelial progenitor cells (EPCs) proliferation, reduces EPCs and macrophages apoptosis, and enhances cholesterol efflux. C, Albumin‐bound S1P has atherosclerotic effect through S1PR2. iNOS, inducible NO synthase; MMP9, matrix metalloproteinase 9; PAI‐1, plasminogen activator inhibitor 1

High‐density lipoprotein (HDL) has been considered as a protective factor for atherosclerosis because of its aid in reverse cholesterol transport from the artery walls to the liver for excretion and protection against inflammation. In view of HDL being good for the body, several drugs are being studied to increase blood HDL levels.^71^ Although HDL harbours a protective effect on CAD, increasing HDL levels is not always beneficial and could have adverse vascular effects since HDL particles are heterogeneous in their lipid and protein composition.^72^ Interestingly, HDL‐associated S1P can impart its beneficial role. HDL‐bound S1P inhibits inducible NO synthase (iNOS) and matrix metalloproteinase 9 (MMP9), both of which take active part in the inflammatory process of atherosclerosis. In spite of that, S1P‐unbound HDL (HNF1a−/−) would lose this enzyme‐inhibiting activity but can restore the anti‐inflammatory property after reloading S1P. Mechanistically, HDL‐S1P works mainly through S1PR2 (Figure 4A),^73^ namely the protective effect of HDL should be essentially attributed to its partner S1P.

Likewise, HDL‐S1P exerts anti‐inflammatory and anti‐atherosclerotic effects on macrophages, which is an important participant in the development of atherosclerosis, including involvement in cholesterol accumulation and plaque formation. Macrophage apoptosis is known to shape atherosclerotic plaque formation throughout all stages of atherosclerosis. Feuerborn et al found that HDL‐S1P inhibits macrophage apoptosis by activating STAT3 and promoting survivin expression through S1PR2/S1PR3 signalling (Figure 4A).^74^ Moreover, endothelial progenitor cells (EPCs) are able to prevent the development of atherosclerosis by repairing endothelial damage and restore monolayer integrity and function, S1P promotes EPCs proliferation and inhibits their apoptosis through S1PR1 and S1PR3 to activate the PI3K/Akt signalling pathway (Figure 4B).^49^ The role of S1PR1 in atherosclerosis can be demonstrated that in vivo, myeloid‐specific S1PR1 deficiency begat accelerated development of atherosclerosis as well as necrotic core formation and the appearance of apoptotic cells within atherosclerotic plaques. On the contrary, S1PR1 selective agonist SEW2871 imparted protection of macrophages from apoptotic damage through the activation of the PI3K/Akt signalling (Figure 4B).^75^ In general, the beneficial effects of S1P against atherosclerosis are multifaceted. Increased plasma S1P is helpful in reducing monocyte adhesion and transport across endothelial layer and minimizing endothelial cell permeability.^22^ Furthermore, S1P signalling is a regulator of glucose and lipid metabolism. After endogenous S1P generated by SphK2 binds to and activates S1PR3, both transcriptional and functional ABCA1 regulatory pathways are active in mediating cholesterol outflow in a positive feedback loop (Figure 4B).^76^

Nevertheless, some studies have suggested that S1P can promote atherosclerosis instead. S1P can promote lymphocyte outflow and activation and thrombus formation,^77^, ^78^, ^79^ thereby partially contributing to the progression of atherosclerosis. The differential biological regulations of S1P correlate with the complex chemistry profile of plasma S1P. It has been demonstrated that circulating S1P has a unique characteristic distribution; approximately two‐thirds of plasma S1P are carried by HDL, followed by albumin. Actually, patients with coronary heart disease have lower total plasma S1P and HDL‐S1P levels, but higher albumin‐bound S1P, than healthy individuals.^77^ Undoubtedly, S1P has a double role in atherosclerosis. On the one hand, albumin‐bound S1P promotes atherosclerosis by increasing the expression of plasminogen activator inhibitor 1 (PAI‐1) through the S1PR2‐Rho/ROCK‐NF‐κB pathway (Figure 4C).^80^ On the other hand, HDL‐bound S1P mainly exerts anti‐atherosclerotic properties by boosting the formation of the S1PR1‐β‐arrestin 2 complex and concomitantly reducing the ability of TNF‐α to activate NF‐κB and ICAM‐1 (Figure 4A).^81^

Based on the information above, S1P and HDL may have adverse health effects when working independently. Only when acting synergistically can they display a protective function. Although the mechanism of S1P in atherosclerosis is complex and multifactorial, determining the S1P chemistry may help clinical management of atherosclerosis, such as developing HDL‐S1P‐increasing pharmacological therapy and nutritional intervention.^82^

## 4 CLINICAL APPLICATION OF S1P‐BASED MODULATORS

There are currently several clinical trials of S1P axis‐targeting modulators for different diseases. Note that most of these modulators are directed to the S1PRs rather than the S1P itself. The representative drug to mention here is fingolimod, which has mainly been used for the treatment of neurodegenerative diseases, such as multiple sclerosis.^2^ Although very promising, its use is limited due to the presence of adverse cardiovascular events. Therefore, fingolimod has been used as a positive control to comparatively evaluate the cardiovascular safety of other S1P‐related agents. For example, compared to fingolimod, amiselimod (MT‐1303) as a selective S1PR1 modulator has been demonstrated to have a better cardiac safety profile in all preclinical, phase I and phase II studies.^83^, ^84^ Of note, certain adverse events produced by S1P‐based therapies, such as lower heart rate, are resulted from their selective binding to putative receptors or non‐selective binding to other receptors and can be influenced by drug dose titrations.^85^ Albeit causing adverse vascular effects, fingolimod was found to lessen vascular inflammation. In patients with hemispheric ischaemic stroke caused by anterior or middle cerebral artery occlusions, combined treatment of fingolimod and alteplase reduced circulating lymphocyte counts and lesion size, attenuated bleeding and reperfusion injury and resultantly improved the clinical outcome of acute ischaemic stroke.^86^ Another clinical trial also confirmed the effective effect of fingolimod on the recovery of neurological function in patients with acute ischaemic stroke.^87^ Additionally, inflammatory response after intracerebral haemorrhage (ICH) leads to perihematomal oedema and secondary brain injury, but fingolimod can relieve oedema and reduce ICH‐related lung infections.^88^ At present, S1P‐related agents are mainly used for immune diseases, and their application in cardiovascular diseases needs more attention and investigation.

## 5 CONCLUSION

Sphingosine 1‐phosphate is a biologically active lipid regulator that is not only involved in the regulation of an array of physiological functions in distinct cell types, but also implicated in the pathogenesis of many diseases and disorders. In this review, we focus on the direct involvement of S1P and S1PRs in the cardiovascular system. Evidently, the regulatory effects of S1P and its receptors in cardiovascular system are multifaceted and sometimes even opposite, depending on the S1P chemistry, SphK isoenzymes, carrier protein, receptor subtypes, downstream effectors, cell types and disease staging, among others. In consideration of these facts, S1P signalling provides an abundance of potential molecular targets for novel therapeutic interventions. There is a great opportunity to improve many cardiovascular diseases through S1P, such as myocardial infarction, myocarditis, vascular injury and atherosclerosis, and however, additional carefulness is needed in developing S1P‐based treatment approaches, which should be selective with less off‐target effects and had better act locally to minimize adverse effects in other organs and systems. As mentioned above, the S1PR1 selective agonist MT‐1303 is safer in cardiac protection than the non‐selective S1PR1 and S1PR3 receptor agonist fingolimod.^83^, ^84^ S1P‐related modulators are being actively and extensively evaluated for the treatment of other diseases, but human‐based studies and cardiovascular disease‐targeting trials are still relatively few, and therefore, more data are needed to prove the availability of S1P‐based therapies, which may provide insights into its future use in cardiovascular diseases.

## CONFLICT OF INTEREST

The authors confirm that there are no conflicts of interest.

## AUTHOR CONTRIBUTION

**Jie Ouyang:** Conceptualization (equal); resources (lead); software (lead); writing – original draft (lead); writing – review and editing (equal). **Zhihao Shu:** Resources (supporting); software (equal); visualization (equal). **Shuhua Chen:** Resources (supporting); visualization (equal). **Hong Xiang:** Resources (supporting); visualization (equal). **Hongwei Lu:** Funding acquisition (lead); resources (supporting); supervision (lead); writing – review and editing (equal).

## Data Availability

The review is exempt from data sharing.
